# Novel Compound MMV1804559 from the Global Health Priority Box Exhibits In Vitro and In Vivo Activity against *Madurella mycetomatis*

**DOI:** 10.3390/ijms25116227

**Published:** 2024-06-05

**Authors:** Jingyi Ma, Kimberly Eadie, Marij Schippers, Ahmed Fahal, Benoît Laleu, Annelies Verbon, Wendy W. J. van de Sande

**Affiliations:** 1 Department of Medical Microbiology and Infectious Diseases, Erasmus MC, University Medical Center Rotterdam, Dr. Molewaterplein 40, 3015GD Rotterdam, The Netherlands; m.jingyi@erasmusmc.nl (J.M.); k.eadie@erasmusmc.nl (K.E.); m.schippers@erasmusmc.nl (M.S.); a.verbon@umcutrecht.nl (A.V.); 2 Mycetoma Research Centre, Khartoum 1115, Sudan; ahfahal@hotmail.com; 3 MMV Medicines for Malaria Venture, 1215 Geneva, Switzerland; laleub@mmv.org

**Keywords:** *Madurella mycetomatis*, mycetoma, eumycetoma, treatment, COVID box, Global Health Priority Box, MMV open access boxes

## Abstract

Objectives: Eumycetoma is a neglected tropical disease (NTD) characterized by subcutaneous lesions and the formation of grains. Attempts to treat eumycetoma involve a combination of antifungal treatment and surgery, although the outcome is frequently disappointing. Therefore, there is a need to identify novel antifungal drugs to treat eumycetoma. In this respect, Medicines for Malaria Venture (MMV) has assembled libraries of compounds for researchers to use in drug discovery research against NTD. Therefore, we screened two MMVOpen compound libraries to identify novel leads for eumycetoma. Methods: A total of 400 compounds from the COVID Box and the Global Health Priority Box were screened in vitro at 100 µM and 25 µM against the most common causative agents of eumycetoma, namely *Madurella mycetomatis* and *Falciformispora senegalensis,* and the resulting IC_50_ and MIC_50_ values were obtained. Compounds with an IC_50_ < 8 µM were identified for possible in vivo efficacy studies using an *M. mycetomatis* grain model in *Galleria mellonella* larvae. Results: Out of the 400 compounds, 22 were able to inhibit both *M. mycetomatis* and *F. senegalensis* growth at 100 µM and 25 µM, with compounds MMV1593278, MMV020335, and MMV1804559 being selected for in vivo testing. Of these three, only the pyrazolopyrimidine derivative MMV1804559 was able to prolong the survival of *M. mycetomatis*-infected *G. mellonella* larvae. Furthermore, the grains in MMV1804559-treated larvae were significantly smaller compared to the PBS-treated group. Conclusion: MMV1804559 shows promising in vitro and in vivo activity against *M. mycetomatis*.

## 1. Introduction

Mycetoma is a neglected tropical disease (NTD) that is associated with more than 90 different infectious agents, including both bacteria (actinomycetoma) and fungi (eumycetoma) [1]. It is characterized by subcutaneous swellings and the formation of grains [2,3] and is mainly endemic in countries with a dry climate, such as Sudan, Senegal, Mexico, and India. The most common causative agents of actinomycetoma are the bacteria *Nocardia brasiliensis, Streptomyces somaliensis,* and *Actinomadura madurae,* which either form white or yellow grains. The most common causative agents of eumycetoma are *Madurella mycetomatis, Falciformispora senegalensis,* and *Trematosphaeria grisea*, which all form black grains [2,3].

The treatment of mycetoma depends on its etiology. Actinomycetoma is treated by a combination of antimicrobial agents, resulting in a cure rate of >95% [4], while eumycetoma is treated with a combination of antifungal agents and surgery, resulting in a cure rate of <26% and an amputation rate of 6.1% [5]. Currently, itraconazole is the drug of choice for treating eumycetoma patients, being given in daily dosages ranging from 200 to 400 mg for at least 6 months. After this time, the lesion is surgically removed [6]. Itraconazole is very active in vitro against the most common causative agent of eumycetoma, with MIC values ranging from 0.003 to 1 µg/mL for *M. mycetomatis,* 0.016 to 0.125 µg/mL for *F. senegalensis,* and from 0.5 to 2 µg/mL for *T. grisea.* For *M. mycetomatis,* an epidemiological cut-off value of 1 µg/mL itraconazole has been reported [7]. However, itraconazole is unable to kill any fungal grains in patients; even after six months of treatment, the grains removed during surgery are still viable when they are cultured on agar [6]. After surgery, patients are treated for at least another 6 months with itraconazole at dosages of 400 mg/day, although the recurrence of fungal grains is still common [5]. Clinically, the main benefit of itraconazole with respect to fungal grains appears to be stimulating the creation of a fibrous capsule around the grains, which makes it easier to excise the complete lesion during surgery [6]. This sub-optimal clinical activity on eumycetoma patients was also previously demonstrated in both *Galleria mellonella* and murine models of infection, where itraconazole (like most other azoles) did not prolong the survival of infected larvae or prevent fungal grain formation in mice [8,9]. Obviously, novel drugs with novel modes of action need to be identified in order to improve current clinical treatment options for eumycetoma.

Therefore, in 2018, we established an Open Source drug discovery program for mycetoma, named MycetOS [10], and subsequently obtained several collections of compounds from the ‘open innovation program’ of Medicines for Malaria Venture (MMVOpen). The MMVOpen program aims to catalyze drug discovery in the field of neglected tropical diseases [11]. The compounds obtained were tested against *M. mycetomatis,* the main causative agent of eumycetoma [1,11], and later against other causative agents of eumycetoma. We also screened three MMV open access boxes: the pathogen box, stasis box, and pandemic response box [10,12]. From those boxes, we identified ravuconazole and olorofim as promising candidates, which were already in phase II trials for other fungal infections [12]. Additionally, the fenarimols and benzimidazoles showed high antifungal efficacy during our in vitro susceptibility testing and in our in vivo *G. mellonella* larvae model, possibly representing potential new classes of antifungal agents that could be chemically optimized to become more effective in penetrating the fungal grain in eumycetoma patients [10,12,13].

Since the start of the MycetOS project in 2018, MMV has launched two new open-access compound libraries, namely the COVID Box and the Global Health Priority Box [14,15,16]. The COVID Box contains 160 marketed compounds in development that possess known or predicted activity against SARS-CoV-2 [15], while the Global Health Priority Box contains 240 compounds that had previously been shown to have activity against (i) drug-resistant malaria (80 compounds), (ii) malaria carriage in various zoonotic vector species (80 compounds), or (iii) various neglected (non-mycetoma) and zoonotic diseases (80 compounds) [16]. Further, as our previous MycetOS research using MMV compound libraries had resulted in potential lead compounds, we hypothesized that screening these new compound libraries could identify additional novel lead compounds. Therefore, in this study, we set out to screen the COVID Box and the Global Health Priority Box from MMV against the two most common causative agents of eumycetoma in order to identify novel classes of compounds that could be further developed as treatments for eumycetoma. We screened these compound libraries in vitro against *M. mycetomatis* and *Falciformispora senegalensis* and then tested the most promising compounds in vivo using our *M. mycetomatis*-infected *G. mellonella* grain larvae model.

## 2. Results

### 2.1. Twenty-Two Compounds Were Able to Inhibit the Growth of Both M. mycetomatis and F. senegalensis in In Vitro Susceptibility Testing

**Figure 1:**
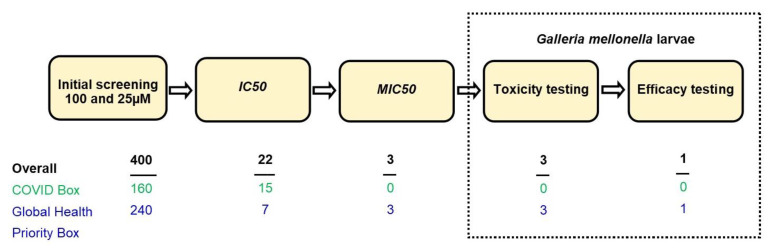
Flow diagram for in vitro and in vivo evaluation of the MMV compounds tested. In total, 400 compounds were tested for growth inhibition of both *M. mycetomatis* and *F. senegalensis* at 100 µM and 25 µM. Fifteen of the 160 compounds present in the MMV COVID Box (green) inhibited the growth of both *M. mycetomatis* and *F. senegalensis.* A total of 7 of the 240 compounds from the MMV Global Health box (blue) were able to inhibit the growth of both fungi. The concentrations at which these 22 compounds were able to achieve a 50% (IC_50_) reduction in fungal growth for *M. mycetomatis* and *F. senegalensis* were determined, with three compounds from the MMV Global Health box achieving IC_50_ at a concentration below 8 µM for *M. mycetomatis*. Additionally, the MIC of these three compounds was determined against eight other *M. mycetomatis* isolates, and all three compounds were able to inhibit the growth of eight additional isolates, namely CBS132419, CBS132588, CBS132589, p1, PARIJS 15580 AL1, SO1, Peru72012, and CBS247.48. After subsequent toxicity and efficacy testing in *G. mellonella* larvae, it was found that one compound, compound MMV1804559, was able to prolong larval survival and, therefore, was considered a new lead compound.

**Figure 2:**
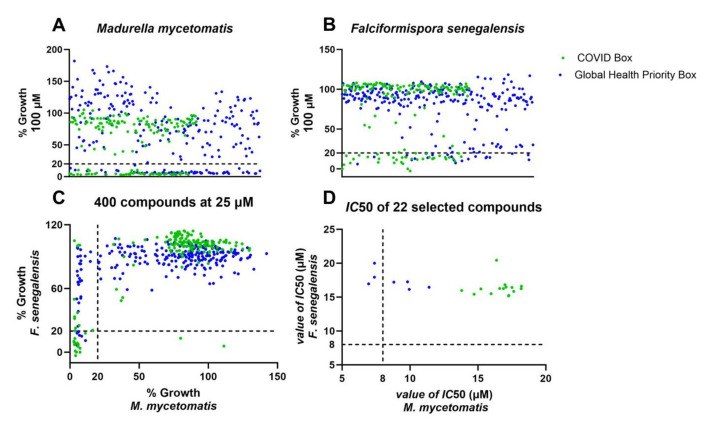
The activities of compounds from the MMV COVID Box and MMV Global Health Priority Box against the two main causative agents of eumycetoma, i.e., *M. mycetomatis* MM55 and *F. senegalensis* CBS197.79. In panel (**A**), the percentage growth of *M. mycetomatis* exposed to 100 µM of each compound is shown. In panel (**B**), the percentage growth of *F. senegalensis* exposed to 100 µM of each compound is shown. Each dot represents a single compound. A green dot indicates a compound that originated from the MMV COVID Box, and a blue dot indicates a compound that originated from the MMV Global Health Priority Box. Growth inhibition was indicated when the percentage growth of fungi was inhibited by ≥80%. (indicated by a dashed line). In panel (**C**), the percentage growth of *M. mycetomatis* (*x*-axis) and *F. senegalensis* (*y*-axis) exposed to a compound concentration of 25 µM is plotted. In total, 22 compounds had a percentage growth inhibition of ≥80% for both *M. mycetomatis* and *F. senegalensis.* In panel (**D**), the IC_50_ values in µM of these 22 selected compounds against *M. mycetomatis* (*x*-axis) and *F. senegalensis* (*y*-axis) are shown. The dashed line shows the 8 µM boundary, i.e., the IC_50_ concentration at which a compound would be selected for in vivo testing.

In this study, we determined the in vitro activity of 160 compounds from the MMV COVID Box and 240 compounds from the MMV Global Health Priority Box (Figure 1). In total, this meant that 400 compounds were tested against both *M. mycetomatis* and *F. senegalensis* at 100 and 25 µM per compound. Of these, 115 compounds inhibited *M. mycetomatis* growth and 59 *F. senegalensis* growth at 100 µM (Figure 2A,B). Of these 115 compounds, 59 also inhibited the growth of *M. mycetomatis* at 25 µM, and 24 inhibited the growth of *F. senegalensis* at 25 µM, as seen in Figure 2C. In total, 22 compounds were active against both eumycetoma fungal species studied. Fifteen of these 22 compounds originated from the MMV COVID Box, and 7 originated from the MMV Global Health Priority Box (4 from the ZND plate and 3 from the MB2 plate) (Table 1).

### 2.2. MMV1593278, MMV020335, and MMV1804559 from the MMV Global Health Priority Box Had an IC_50_ below 8 µM for M. mycetomatis

**Figure 3:**
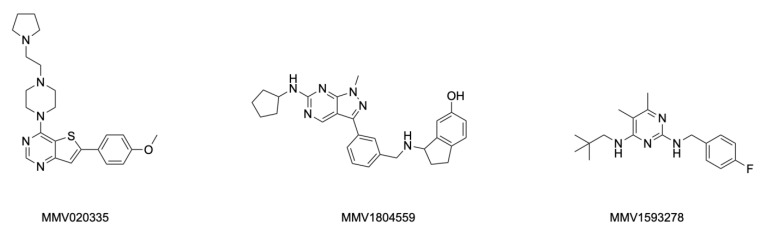
Chemical structures of compounds MMV020335, MMV1804559, and MMV1593278 from the MMV Global Health box with an IC_50_ < 20% for *M. mycetomatis*.

In order to rank the in vitro activity of the 22 selected compounds showing ≥80% fungal growth inhibition, IC_50_ values were obtained against both *M. mycetomatis* and *F. senegalensis*. As can be seen in Figure 2D, three of these 22 compounds had an IC_50_ below 8 µM for *M. mycetomatis* but not for *F. senegalensis.* The IC_50_ of these compounds ranged between 16.95 µM and 20.01 µM for *F. senegalensis* (Figure 2D, Table 1). These three compounds (MMV1593278, MMV020335, and MMV1804559) originated from the MMV Global Health Priority Box. The chemical structures of these three compounds are shown in Figure 3. For these three compounds, the activities against eight additional *M. mycetomatis* isolates were determined and are shown in Table 2. For MMV1593278, the MICs ranged from 8 µM to 16 µM, and 8 µM was needed to inhibit the growth of 50% of the *M. mycetomatis* isolates tested. For MMV020335, the MICs ranged from 16 µM to 32 µM, and 32 µM was needed to inhibit the growth of 50% of the *M. mycetomatis* isolates tested. For MMV1804559, the MICs ranged from 8 µM to 32 µM, and 8 µM was needed to inhibit the growth of 50% of the *M. mycetomatis* isolates tested.

### 2.3. In Vivo Efficacy of MMV020335, MMV1593278 and MMV1804559 from the MMV Global Health Priority Box

**Figure 4:**
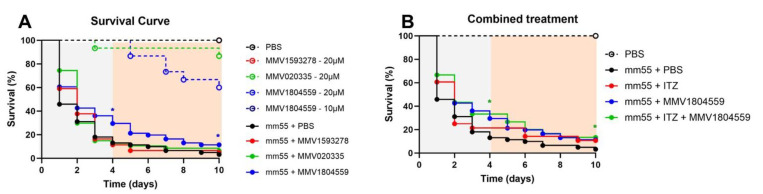
Survival curve of treatment therapy against *M. mycetomatis*-infected larvae using three selected compounds. In panel (**A**), the toxicity (dashed lines) and efficacy (solid lines) of MMV1593278 (red), MMV020335 (green), and MMV1804559 (blue) in *G. mellonella* larvae are shown. PBS was used as a growth control (black). Toxicity testing was performed in uninfected larvae at 20 µM/larva for all three compounds and 10 µM/larva for MMV1804559 (dark blue). Efficacy testing was performed using 20 µM/larva for MMV1593278 and MMV020335 and at 10 µM/larva for MMV1804559 in *M. mycetomatis*-infected larvae. All treated groups were compared with the PBS group via the Log-Rank test. Only MMV1804559 prolonged the survival of *M. mycetomatis*-infected larvae after 4 days (Log-Rank, *p* = 0.0223) and after 10 days (Log-Rank, *p* = 0.0313). Prolonged survival is indicated with an * at day 4 and day 10. No enhanced survival was noted for MMV1593278 and MMV020335. In panel (**B**), the efficacy of the combination of MMV1804559 with itraconazole (ITZ) is shown. The survival curves of larvae treated with PBS (black), MMV1804559 at 10 µM/larva (blue), 5.7 mg/kg ITZ (red), and the combination of MMV1804559 at 10 µM/larvae with 5.7 mg/kg ITZ (green) are shown. Significantly enhanced survival is indicated with an * and observed for larvae treated with MMV1804559 only (Log-Rank, *p* = 0.0313), the combination of MMV1804559 and ITZ after 4 days (Log-Rank, *p* = 0.0227), and the combination of MMV1804559 and ITZ after 10 days (Log-Rank, *p* = 0.0465). * indicates a *p*-value between 0.01 and 0.05.

Compounds MMV1593278 and MMV020335 were found to be non-toxic to *G. mellonella* larvae at 20 µM/larva. However, compound MMV1804559 significantly decreased the survival of healthy, uninfected *G. mellonella* larvae at 20 µM/larva and was therefore considered toxic. Lowering the concentration of MMV1804559 to 10 µM/larvae resolved the toxicity, as no healthy *G. mellonella* larvae died when treated with this concentration (Figure 4A). Compounds MMV1593278, MMV020335, and MMV1804559 were used in in vivo therapeutic efficacy experiments in *M. mycetomatis*-infected larvae, as they exhibited in vivo IC_50_ therapeutic efficacy values below 8 µM against this fungus. Results are shown in Figure 4A, where enhanced survival was noted in *M. mycetomatis*-infected larvae when treated with 10 µM/larva MMV1804559 (Log-Rank, *p* = 0.0313). To determine if combining MMV1804559 with 5.7 mg/kg itraconazole (ITZ) could further enhance therapeutic efficacy, a combination treatment was studied. As can be seen in Figure 4B, combining MMV1804559 with ITZ resulted in enhanced survival (Log-Rank *p* = 0.0465) when compared to the PBS-treated group. However, no enhanced survival was noted compared to the MMV1804559-only treated group (Log-Rank, *p* = 0.2814).

### 2.4. Smaller Grains Were Observed in Larvae Treated with MMV1804559

**Figure 5:**
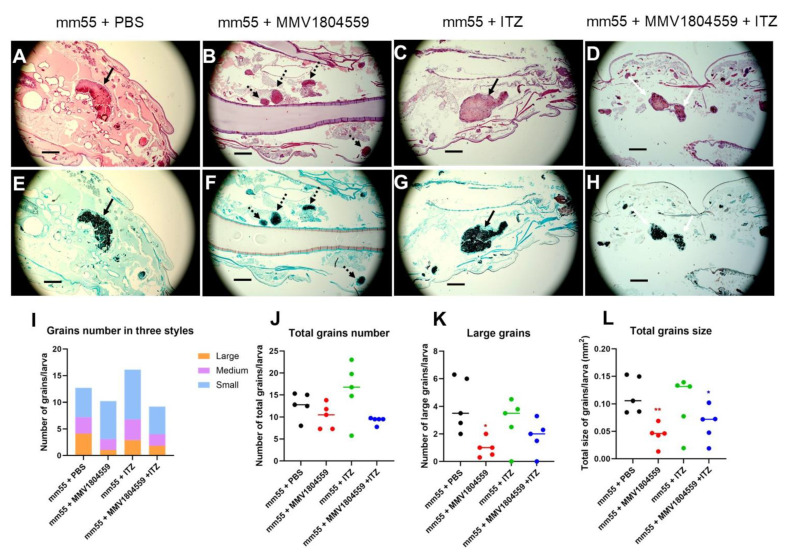
Grains in histology slides of four treatment groups in *M. mycetomatis*-infected larvae. In this figure, H&E-stained (panels (**A**–**D**)) and Grocott-stained (panels (**E**–**H**)) *M. mycetomatis* grains formed in *G. mellonella* larvae are shown. Slides were prepared after 72 h of infection and treatment with PBS (panels (**A**) and (**E**)), 5.7 mg/kg ITZ treatment (panels (**B**) and (**F**)), 10 µM/larva MMV1804559 treatment (panels (**C**) and (**G**)), or the combination of both (panels (**D**) and (**H**)). The scale bars stand for 250 µm in each image. The grains were grouped into large (black arrow), medium (white arrow), and small (dashed arrow) grains. The number of grains that were classified as large (orange), medium (purple), and small (blue) in each slide is depicted in panel (**I**). The Mann–Whitney U-test was used to compare the difference in the total number of grains/larva (panel (**J**)), the total number of large grains (panel (**K**)), and the total size of the grains (panel (**L**)). No significant difference in total grain number (panel (**J**)) was noted. However, a significantly lower number of large grains was noted in larvae treated with MMV1804559 (Mann–Whitney, *p* = 0.0159) (Panel (**K**)). This also resulted in a significantly lower total grain size for larvae treated with MMV1804559 (red) (Mann–Whitney, *p* = 0.0079) or the combination MMV1804559 with ITZ (blue) (Mann–Whitney, *p* = 0.0317) (Panel (**L**)). Statistical significance is displayed as * (0.01 < *p* < 0.05) and ** (0.0001 < *p* < 0.01).

To determine the effect of MMV1804559 on grain formation, histology slides were prepared for *M. mycetomatis*-infected larvae treated with PBS, 10 µM MMV1804559, 5.7 mg/kg ITZ, or a combination therapy of 10 µM MMV1804559 and 5.7 mg/kg ITZ. As can be seen in Figure 5A, mature *M. mycetomatis* grains were formed on day 3. When larvae were treated with 10 µM/larva of MMV1804559, the grains inside the larvae were much smaller (Figure 5B, Table 3). This was also noted when larvae were treated with the combination ITZ and MMV1804559 (Figure 5D, Table 3), but not when larvae were treated with ITZ alone (Figure 5C, Table 3). Despite their smaller size, there was no other striking difference in the morphology of the grains (Figure 5A–D). Additionally, for all grains, cement material was noted between the fungal hyphae, as well as a capsule surrounding the grains. To quantify the difference in grain size, we calculated the number of grains at each grain size. As can be seen in Figure 5I, there were differences between the different treatment groups. The total number of grains (Figure 5J, Table 3) remained stable, but the number of large grains (Figure 5K, Table 3) (Mann–Whitney, *p* = 0.0159) and the average size of the grain (Figure 5L, Table 3) (Mann–Whitney, *p* = 0.0079) were lower in the MMV1804559-treated group compared to the PBS-treated group. Also, the combination of 10 µM/larva MMV1804559 and 5.7 mg/kg ITZ resulted in significantly smaller grains compared to PBS-treated larvae (Mann–Whitney, *p* = 0.0317). This was not the case when larvae were treated with ITZ alone.

## 3. Discussion

In this study, we screened two MMV open-access boxes as part of our Mycetoma Open Source drug discovery program (MycetOS) and identified MMV1804559 as a potential new lead compound [10,12].

MMV1804559 inhibited the growth of *M. mycetomatis* with an IC_50_ of 6.95 µM and an MIC_50_ of 8 µM. Furthermore, MMV1804559 treatment prolonged the survival of *M. mycetomatis*-infected larvae and resulted in smaller grains in the *G. mellonella* larva model of infection. Although very little has been published on MMV1804559, from its chemical structure, it can be seen that MMV1804559 is a pyrazolopyrimidine derivative. A subsequent sub-structure search in SciFinder^®^ identified that close analogs of MMV1804559 have been developed as either Mer tyrosine kinase (MerTK) inhibitors [17,18,19,20,21] or dual-specificity tyrosine-regulated kinase 1 (DYRK1) inhibitors [22]. However, Mer is a receptor tyrosine kinase, and tyrosine kinases are not present in most fungal genomes [23]. Therefore, we hypothesized that the activity of MMV1804559 might occur through the fungal DYRK1. In *Aspergillus* spp., *Candida albicans, Cryptococcus* spp., and *Schizosaccharomyces pombe,* two DYRKs are encoded in their genomes, namely *yak1* and *pom1* [24]. In the *M. mycetomatis* genome, a homolog of *pom1* (KXX76507) was found. Although there is no published research on the deletion of these specific genes in *M. mycetomatis*, it was noted that deleting both *yak1* and *pom1* in *C. albicans* completely blocked filamentation [24]. Additionally, in *Aspergillus fumigatus*, YakA was associated with septal plugging, which led to abrogated growth [25]. This effect on the filamentation in the fungi was also noted in in vivo animal models. For example, in an in vivo *C. albicans* dermatitis model, *yak1* deletion mutants no longer produced polarized *C. albicans* growth but appeared to block morphogenesis in yeast cells [24]. In an *A. fumigatus* lung model, the *A. fumigatus* lesion size was much smaller when mice were infected with an *A. fumigatus yakA* deletion mutant compared to a wild-type isolate [25]. Both Yak1 and YakA in *C. albicans* and *A. fumigatus* could also be inhibited by beta-carboline compounds [24,25,26]. In *A. fumigatus,* these compounds blocked the recruitment of YakA to the septal pore [25], while in *C. albicans*, these compounds blocked biofilm formation in an ex vivo model of vaginal candidiasis and filamentation in the *C. albicans* dermatitis model [24,26]. Although pyrazolopyrimidines were not tested specifically as DYRK inhibitors in *Candida* species, it was established that pyrazolopyrimidines have antifungal activity against *Candida* [27]. In the set of compounds tested in this study, another pyrazolopyrimidine derivative, MMV022478, was active against *Candida albicans, Candida auris,* and *Candida glabrata* [28]. Furthermore, MMV022478 was also able to inhibit *M. mycetomatis* with an IC_50_ of 2.95 µM and prolong larval survival [10]. However, although MMV1804559 decreased the number and size of *M. mycetomatis* grains formed, MMV022478 did not [10]. *Pom1* deletion mutants have not yet been studied in in vivo models. Taken together, these results indicate that pyrazolopyrimidines should be studied further as potential drug candidates for the treatment of eumycetoma.

Since the literature searches showed that close structural analogs of MMV1804559 were developed as human MerTK and DYRK1 inhibitors, it is likely that MMV1804559 not only inhibited fungal DYRKs but could have also inhibited the *G. mellonella* MerTK or DYRK1 kinases. Indeed, at 20 µM/larva, MMV1804559 showed toxicity in uninfected *G. mellonella*, which was also demonstrated in HepG2 cells. In these cells, a concentration of 3 µM MMV1804559 reduced cell viability by 50%. In humans, Mer is a receptor tyrosine kinase that functions as an innate immune system checkpoint in macrophages [23,29]. MerTK is a marker of anti-fibrotic macrophages, is important in the clearance of apoptotic cells, and induces specialized pro-resolving mediators, which results in the clearance of inflammation [30,31,32]. DYRKs play indispensable roles in signal transduction, cellular differentiation, and cell cycle control, as well as in inflammation. In THP-1 macrophages, the inhibition of DYRK1B resulted in a pro-inflammatory cytokine release [33].

Treatment with MMV1804559 resulted in significantly smaller grains in our in vivo *M. mycetomatis* grain model in *G. mellonella.* Unlike mammals, *G. mellonella* only has an innate immune system, and grain formation in *G. mellonella* is a four-step process in which both the fungus and host play a role [34,35]. Therefore, inhibiting MerTK, DYRK1, or both would most likely result in enhanced inflammation in the *G. mellonella* host. However, in our treated *M. mycetomatis*-infected larvae, no evidence of enhanced inflammation was observed. Therefore, as MMV1804559 had a clear effect on inhibiting the growth and grain formation in *M. mycetomatis,* the compound is most likely to exhibit a novel mode of action against this eumycetoma causative agent. Further, although the compound showed toxicity in our in vivo *G. mellonella* model, future investigations into improved compound-fungal structure-activity relationships (SAR) could promote the design of more analogs of this parent molecule that display increased fungal activity but with less toxic activity to the host. For example, such an approach has already proven successful for the compound MMV024478, where two structural modifications of this pyrazolopyrimidine not only increased its efficacy against the fungus *Candida auris* but also decreased its toxicity [28]. Thus, before being able to study the efficacy of this drug in a murine model, novel derivatives of the molecule need to be made that are more selective towards the fungus and less toxic for the mammalian host. The Mycetoma Open Source drug discovery project, MycetOS (https://github.com/OpenSourceMycetoma; accessed on 1 February 2024), is a suitable platform for future research in this area.

## 4. Materials and Methods

### 4.1. Strains

The MMV COVID Box and Global Health Priority Box compound libraries were initially screened against *M. mycetomatis* strain MM55 and *F. senegalensis* strain CBS197.79. MM55 was originally isolated at the Mycetoma Research Centre in Sudan in 1999 and maintained at ErasmusMC [36]. CBS197.79 was obtained from the Westerdijk Fungal Biodiversity Center in The Netherlands. To ensure that the activity was not confined to these two isolates, the most promising compounds were also screened against a further eight different *M. mycetomatis* isolates, namely, CBS132419, CBS132588, and CBS132589 (all originating from India), p1 (originating from Mali), PARIJS 15580 AL1 (originating from Algeria), SO1 (originating from Somalia), Peru72012 (originating from Peru), and CBS247.48 (country of origin unknown) to determine the concentrations that inhibited 50% of all isolates tested (MIC_50_). The CBS isolates were originally obtained from the Westerdijk Fungal Biodiversity Center in Utrecht. All isolates were identified to the species level by sequencing the internally transcribed spacer (ITS) region and maintained in the Erasmus MC laboratory on Sabouraud Dextrose agar plates (BD, Erembodegem, Belgium) [37].

### 4.2. Compound Boxes

The COVID Box and the Global Health Priority Box were kindly donated by MMV. The COVID Box consisted of two plates of 80 compounds, each with known or predicted activity against SARS-CoV-2 [15]. The Global Health Priority Box consisted of 240 compounds divided over three plates, each containing 80 compounds. The first plate consisted of 80 compounds with confirmed activity against drug-resistant malaria; the second plate consisted of 80 compounds with activity against neglected (non-mycetoma) and zoonotic diseases; and the third plate consisted of 80 compounds with activity against various zoonotic vector species, as demonstrated in the Innovative Vector Control Consortium (IVCC) [16]. Therefore, a total of 400 compounds were screened for activity against *M. mycetomatis* and *F. senegalensis* (Supplementary File S1).

### 4.3. In Vitro Susceptibility Assay

To determine the activity of the 400 compounds against the fungal isolates, an initial in vitro screening at concentrations of 100 µM and 25 µM was performed. For this, strains were transferred from Sabouraud culture plates to colorless RPMI 1640 working medium, which contained 0.35 g/L L-glutamine (Gibco, ThermoFisher, Bleiswijk, The Netherlands) and 1.98 mM 4-morpholinepropane sulfonic acid (MOPS; Sigma-Aldrich, Zwijndrecht, The Netherlands). The suspension was then sonicated for 10 s at 10 µm (Soniprep 150 Plus, Medical and Scientific Equipment, Cholet, France) to obtain hyphal fragments, and the resulting suspension was incubated for seven days at 37 °C, after which time the mycelia were harvested by centrifugation at 3400 rpm for 5 min. The harvested mycelium was again sonicated to obtain hyphal fragments, and a working hyphal suspension of 68–72% transmission at 660 nm (Novaspec II; Pharmacia Biotech, Uppsala, Sweden) was prepared in a colorless RPMI working medium. One µL of the diluted compound and 100 µL of hyphal suspension were added to the wells of a round-bottom 96-well plate (Costar 3799, Fisher Scientific, Breda, The Netherlands).

For M. mycetomatis, the plates were then incubated for seven days at 37 °C, after which time 10 µL of ready-to-use viability dye was added (i.e., 3-(4,5-dimethylthiazol-2-yl)-5-(3-carboxymethoxyphenyl)-2-(4-sulfophenyl)-2H-tetrazolium inner salt (MTS)) from the CellTiter 96^®^ AQueous One Solution Cell Proliferation Assay (G3581, Promega, Leiden, The Netherlands). After 2 h incubation at 37 °C, 100 µL of supernatant was transferred to the well of a flat-bottomed plate, and the color intensity was measured at 490 nm using an Epoch 2 (BioTek, Santa Clara, CA, USA) microplate reader.

For *F. senegalensis*, 20 µL of 0.15 mg/mL resazurin was added, after which the plates were incubated for seven days at 37 °C. After seven days, 100 µL of supernatant was transferred to the wells of a flat-bottomed plate, and color intensity was measured at 600 nm using an Epoch 2 (BioTek, Santa Clara, CA, USA) microplate reader. A negative control (NC), consisting of only the culture medium and the viability dye, was used to correct the background signal.

Finally, the metabolic activity of the treated isolate was compared to the metabolic activity of the growth control (GC—no compounds added to the isolates) corrected by the NC using the following formulas [38]:

Complete growth inhibition (MIC) was assumed if a reduction in metabolic activity of >80% was measured. If compounds showed complete growth inhibition at both 100 µM and 25 µM for both species, the IC_50_ was determined by repeating the assay using a two-fold dilution of the compounds, ranging from 0.03 to 16 µM. The minimal inhibitory concentrations (MICs) against additional isolates of the fungal species used were determined using the identical dilution series.

### 4.4. Toxicity of Compounds in Galleria Mellonella Larvae

The toxicity of the compounds with promising in vitro activity was determined in five instar *Galleria mellonella* larvae (Bigbig SA.G.IP larvae, forelshop.be, Baal, Belgium). For this, compounds were diluted in 20 µM/20 µL PBS. Each compound was injected into a group of 15 uninfected, healthy larvae via the pro-leg using an insulin 29G U-100 needle (BD diagnostics, Erembodegem, Belgium). Larvae survival was monitored for 10 days. If a significantly decreased larval survival (compared to the PBS-treated larvae) or an overall larval survival <80% (compared to injection at time 0) was observed, the compound was considered toxic. If toxicity was observed, compound concentrations of 10 µM/20 µL PBS were used.

### 4.5. In Vivo Grain Model in Galleria Mellonella Larvae

A previously described in vivo eumycetoma grain model involving *M. mycetomatis* and the larvae of the invertebrate *G. mellonella* was used for in vivo compound library screening [8]. To determine the efficacy of the selected compounds, larvae were first infected with 4 mg of *M. mycetomatis* per larva. To obtain this inoculum, *M. mycetomatis* strain MM55 was cultured for two weeks at 37 °C in RPMI 1640 working medium containing 0.3 g/L L-glutamine, 20 mM MOPS, and 100 mg/L chloramphenicol. After two weeks, the mycelia were harvested by vacuum filtration through a 0.22 µm filter (Nalgene, Abcoude, The Netherlands). The resulting wet-weight mycelium was weighed and then sonicated for 2 min at 10 microns. The resulting homogeneous suspension was washed once in PBS and further diluted to generate an inoculum of 100 mg/mL *M. mycetomatis*. Forty microliters of fungal suspension were then injected into the last left pro-leg of a larva using an insulin 29G U-100 needle, resulting in the inoculation of 4 mg *M. mycetomatis*/larva. Next, five infected larvae were placed in a Petri dish containing Whatmann paper, which was incubated at 37 °C for 10 days. For compound testing, 15 larvae were treated with either PBS (negative control), itraconazole 5.7 mg/kg (positive control—Janssen Pharmaceuticals, Beerse, Belgium), MMV1593278 at 20 μM/larva, MMV02033520 at 20 μM/larva, MMV1804559 at 10 μM/larva, or a combination of 5.7 mg/kg itraconazole and 10 μM MMV1804559/larva. Each experiment was performed in triplicate. In each larva, each compound was injected three times with an insulin 29G U-100 needle, namely 4, 28, and 52 h after *M. mycetomatis* infection. Per injection, a different pro-leg was used. Larvae were monitored for ten days after compound administration, and their survival was recorded on a daily basis. Survival rates were compared on days four and ten using the Log-Rank test. An initial starting time for compound administration of 4 h post-infection was chosen as fungal grains were already visible in infected larvae at this time point.

### 4.6. Histology

Larvae were infected with *M. mycetomatis* and treated 4, 28, and 52 h after infection, as described above. At 96 h after infection, larvae were injected with 100 µL of 10% buffered formalin and transferred to containers containing 10% buffered formalin. After 24 h incubation in formalin, the larvae were dissected into two parts. Both parts of the larvae were embedded in paraffin, and sections of 5 µm were prepared. The sections were stained with hematoxylin and eosin (H&E) and Grocott methanamine silver and observed under a Canon EOS70D camera (Canon Inc., Amstelveen, The Netherlands) by two independent researchers to avoid individual bias. Every slide was visualized on EOS Utility software version 3.12.30 (Canon Inc., Amstelveen, The Netherlands) at 40× magnification. Grains were categorized into large, medium, or small sizes using an enlargement display frame of 250 µm and 160 µm (present in the Live View Shooting mode) and manually counted under a light microscope mounted using a Canon EOS70D camera. Counts were performed by two independent scientists, as previously described [39]. The sum of all large, medium, and small grains present in larvae was used to represent the total number of grains in the larvae [39]. To estimate the total size of grain present in the larvae, the sum of all grains in a larva was multiplied by the minimum size of their respective category (large: 0.02 mm^2^, medium: 0.01 mm^2,^ and small: 0.005 mm^2^) [39]. The sum of the grain sizes of the three categories together was considered the total grain size. For each treatment group, five larvae were analyzed.

### 4.7. Data Analysis

A Log-Rank test was performed to compare the survival rate between the different compound treatment groups and the PBS-treated group. The Mann–Whitney test was used to calculate the difference in grain number and size. All calculations were performed in GraphPad Prism 10 (Dotmatics, Boston, MA, USA). A *p*-value of <0.05 was considered significant.

## 5. Conclusions

In conclusion, compound MMV1804559 from the MMV Global Health Priority box exhibits promising in vitro and in vivo activity against the eumycetoma-causing fungus *M. mycetomatis*. Further, its pronounced effect on fungal grains during *M. mycetomatis* infection of *G. mellonella* makes MMV1804559 an attractive compound for further study as part of our Open Source drug discovery program, MycetOS.

## Figures and Tables

**Table 1:** IC_50_ values of the 22 most potent compounds against both *M. mycetomatis* and *F. senegalensis*.

Compound ID	Location	Compound Name or Chemical Class	IC_50_ of mm55 (µM)	IC_50_ of CBS197.79 (µM)
**Global Health Priority Box**				
MMV1804559	Global Health Priority Box MB2 plate	Pyrazolo [3,4-d]pyrimidine	6.95	16.95
MMV1593278	Global Health Priority Box ZND plate	2,4-Diaminopyrimidine	7.38	17.94
MMV020335	Global Health Priority Box MB2 plate	Thieno[3,2-d]pyrimidine	7.39	15.00
MMV1542799	Global Health Priority Box ZND plate	Purine-2,8-diamine	8.81	17.20
MMV1545674	Global Health Priority Box ZND plate	2,6-Diaminoimidazo[4,5-c]pyrimidine	9.83	17.26
MMV1542798	Global Health Priority Box ZND plate	2,8-Diaminopurine	9.95	16.15
MMV024638	Global Health Priority Box MB2 plate	Pyrrolo[2,3-c]pyridine	11.40	16.45
**COVID Box**				
MMV637229	COVID Box plate-B	Clemastine	13.80	15.97
MMV003461	COVID Box plate-A	Niclosamide	14.72	15.43
MMV003162	COVID Box plate-A	Astemizole	15.22	16.21
MMV1804244	COVID Box plate-A	Triparanol	15.97	15.51
MMV000016	COVID Box plate-B	(+)-Mefloquine	16.37	20.44
MMV001428	COVID Box plate-B	Thiethylperazine	16.60	16.28
MMV638007	COVID Box plate-B	Toremifene	16.88	16.26
MMV001871	COVID Box plate-A	Chlorpromazine	17.00	16.83
MMV001829	COVID Box plate-A	Fluphenazine	17.07	16.48
MMV1804190	COVID Box plate-A	Bemcentinib	17.25	15.18
MMV001681	COVID Box plate-B	Fluspirilene	17.28	15.26
MMV1580167	COVID Box plate-B	Ponatinib	17.51	16.42
MMV1580492	COVID Box plate-A	Ozanimod	17.64	15.84
MMV892669	COVID Box plate-B	Desmethyl ferroquine	18.19	16.24
MMV690733	COVID Box plate-B	Osimertinib	18.21	16.61

Note: Ranked by IC_50_ value against *M. mycetomatis* MM55.

**Table 2:** MIC_50_ values of three selected compounds from the Global Health Priority Box.

Strain	MMV1593278 MIC (µM)	MMV020335 MIC (µM)	MMV1804559 MIC (µM)
CBS132419	8	16	8
CBS132588	8	32	8
CBS132589	8	32	8
p1	16	16	32
PARIJS 15580 AL1	8	32	16
SO1	8	16	16
Peru72012	8	32	16
CBS247.48	8	16	8
MM55	8	32	8
MIC_50_	8	32	8

**Table 3:** Data analysis of grain number and size in *M. mycetomatis*-infected larvae treated with single and combined therapy.

	Number	In Vivo Significance (*p*-value)		AVERAGE Grain Number Per Size			Median of Total Grain Number	Mann–Whitney *p*-Value	Median of Large Grain Number	Mann–Whitney *p*-value	Median of Total Grain Size	Mann–Whitney *p*-Value
		Day 4	Day 10	Large (STDEV)	Medium (STDEV)	Small (STDEV)	Average (STDEV)		Average (STDEV)		Average (STDEV)	
Control												
PBS	5			4.10 (1.93)	3.10 (0.63)	5.50 (2.57)	12.70 (2.91)		4.10 (1.93)		0.12 (0.03)	
Global Health Priority Box												
MMV1593278	5	NS	NS	/	/	/	/	/	/	/	/	/
MMV020335	5	NS	NS	/	/	/	/	/	/	/	/	/
MMV1804559	5	Increase survival (0.0223 *)	Increase survival (0.0313 *)	0.95 (0.67)	2.10 (2.15)	7.05 (1.24)	10.10 (2.85)	NS	0.95 (0.67)	0.0159 *	0.04 (0.02)	0.0079 **
ITZ	5	NS	NS	2.85 (1.75)	3.85 (1.92)	9.03 (3.59)	16.00 (6.52)	NS	2.85 (1.75)	NS	0.10 0.05)	NS
MMV1804559 + ITZ	5	Increase survival (0.0227 *)	Increase survival (0.0465 *)	1.80 (1.19)	2.20 (1.30)	5.20 (1.69)	9.20 (0.82)	NS	1.80 (1.19)	NS	0.06 (0.03)	0.0317 *

Note: significant differences are displayed as NS (Not significant, *p* > 0.05); * (0.01 < *p* < 0.05) and ** (0.001 < *p* < 0.01).

## Data Availability

Data are contained within the article or Supplementary Materials.

## Supplementary Materials

The following supporting information can be downloaded at: https://www.mdpi.com/article/10.3390/ijms25116227/s1.
